# Severe Primary Hypothyroidism in a Child Presenting as a Pituitary Tumour: A Case Report

**DOI:** 10.7759/cureus.94880

**Published:** 2025-10-18

**Authors:** Victoria Leal, Shokery Awadalla

**Affiliations:** 1 Pediatric Endocrinology, Fundacion Universitaria de Ciencias de la Salud, Bogotá, COL

**Keywords:** low free t4, pediatric hypothyroidism, pituitary hyperplasia, pituitary tumour, thyroid-stimulating hormone (tsh)

## Abstract

Primary hypothyroidism in children often manifests as short stature, and if untreated, it can result in pituitary hyperplasia due to chronic thyrotropin-releasing hormone (TRH) drive resulting from the loss of negative feedback by low circulating thyroid hormones, potentially leading to unnecessary surgery. We report the case of a nine-year-old patient with a persistent headache with a suspected diagnosis of a pituitary mass. Blood tests revealed severe hypothyroidism (extremely elevated thyroid-stimulating hormone (TSH) and suppressed free thyroxine (T4). The initial MRI led to a misdiagnosis of pituitary adenoma. Reversible pituitary hyperplasia completely regressed following levothyroxine therapy. This case highlights the relevance of considering hypothyroidism when evaluating pituitary masses. Thyroid function should always be assessed before considering pituitary surgery in children.

## Introduction

Thyroid hormone secretion and function are regulated through the hypothalamic-pituitary-thyroid axis. Thyrotropin-releasing hormone (TRH), secreted by the hypothalamus, stimulates the pituitary to produce thyroid-stimulating hormone (TSH). TSH has trophic and functional effects on the thyroid gland, leading to the synthesis and secretion of thyroxine (T4) and triiodothyronine (T3), which in turn exert inhibitory feedback on both the hypothalamus and the pituitary. The main cause of primary hypothyroidism worldwide is iodine deficiency and Hashimoto's thyroiditis in iodine-sufficient areas. [1].

Hypothyroidism has an insidious onset, and symptoms in children are often nonspecific; however, growth failure is often the most prominent and clinically important feature. A decrease in thyroid hormones reduces negative feedback, leading to elevated TRH; if prolonged, this can result in persistent elevation in TRH levels and pituitary hyperplasia, which may be misinterpreted as pituitary adenoma [2,3].

Pituitary hyperplasia, though rare, may occur as an unanticipated manifestation of long-standing primary hypothyroidism, sometimes mimicking a pituitary adenoma. If the diagnosis and treatment are delayed, misdiagnosis as a pituitary tumour can lead to unnecessary surgery and hypopituitarism [4]. We present a case of pituitary enlargement secondary to primary hypothyroidism, initially misinterpreted as a pituitary adenoma, with discussion and recommendations that address the global burden of delayed diagnosis in resource-limited settings.

This article was previously presented as a meeting abstract at the 2024 Endocrine Society's (ENDO) Annual Scientific Meeting on June 1, 2024.

## Case presentation

**Figure 1:**
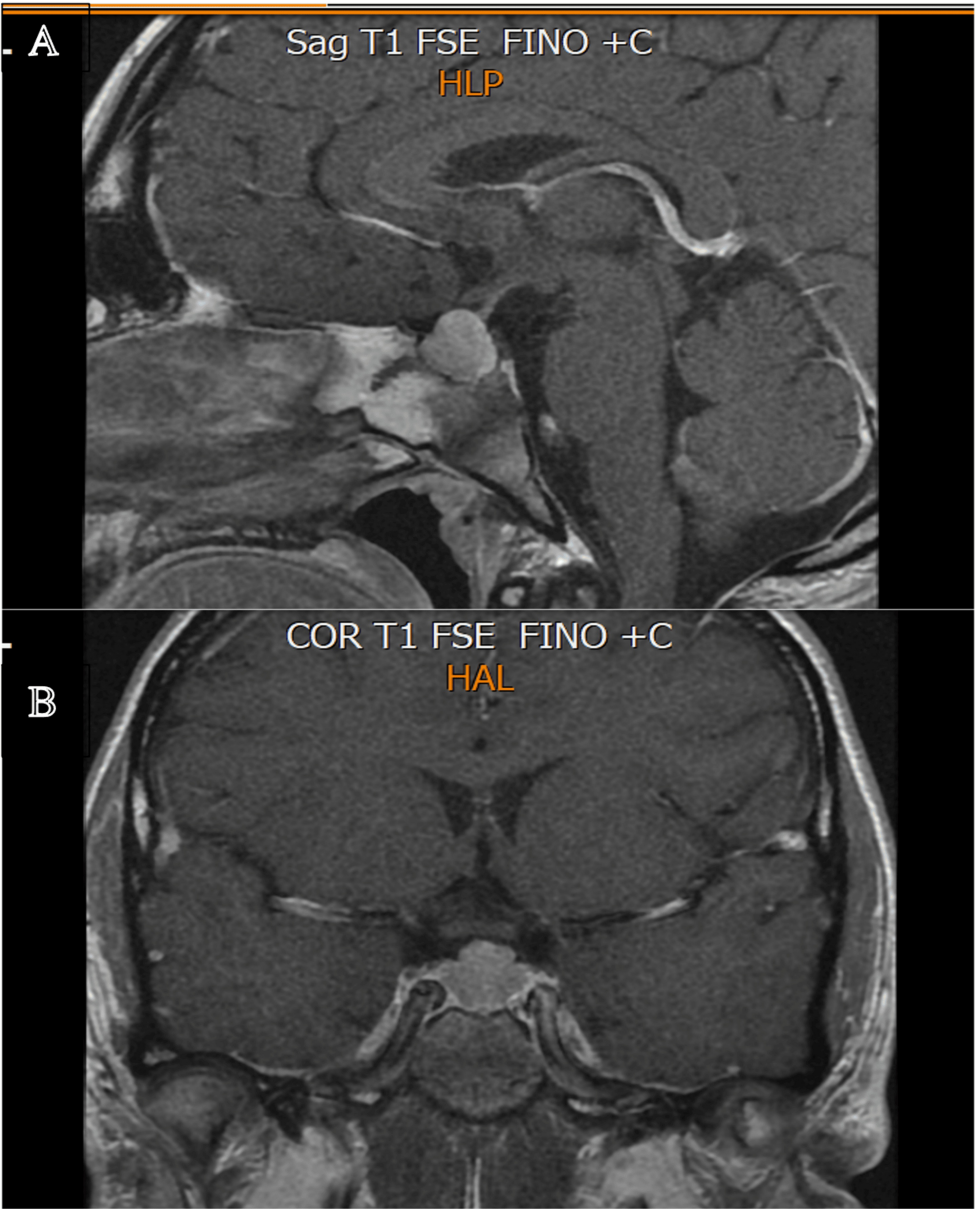
MRI images showing pituitary enlargement, an expansive intrasellar and suprasellar lesion measuring 14×13×13 mm, were initially interpreted as a pituitary adenoma. (A) sagittal view; (B) coronal view

A nine-year-old male patient, previously asymptomatic, presented to the emergency department with a headache that had persisted for one month, without any other symptoms. The patient's history was unremarkable, with no family history of thyroid disease. The physical examination showed no neurological findings and, crucially, no goitre. The only notable exception was the severe short stature (height: 117 cm, standard deviation score (SDS): 3.0), with a weight of 25 kg (SDS: .1). Pubertal development was Tanner stage I (pubic and testicular), and there were no subtle characteristics of hypothyroidism, such as dry skin or bradycardia. Laboratory tests, including complete blood count and blood glucose, were also normal. Given the persistence of the headache, a brain MRI was performed, which revealed pituitary enlargement that was interpreted as a pituitary adenoma. A dedicated MRI of the sella turcica showed similar findings, with pituitary enlargement again interpreted as a pituitary adenoma (Figure 1).

The pediatric endocrinology department evaluated pituitary function. TSH, free T4, and cortisol levels were measured. Results showed markedly elevated TSH (936 mIU/L) and suppressed free T4 (< 0.4 ng/dL), while cortisol levels were within the normal range (16.1 µg/dL AM). The case was discussed with the neurosurgery department, and given the markedly elevated TSH, thyroid hormone replacement therapy was initiated. Re-evaluation was planned once thyroid function had normalised.

**Figure 2:**
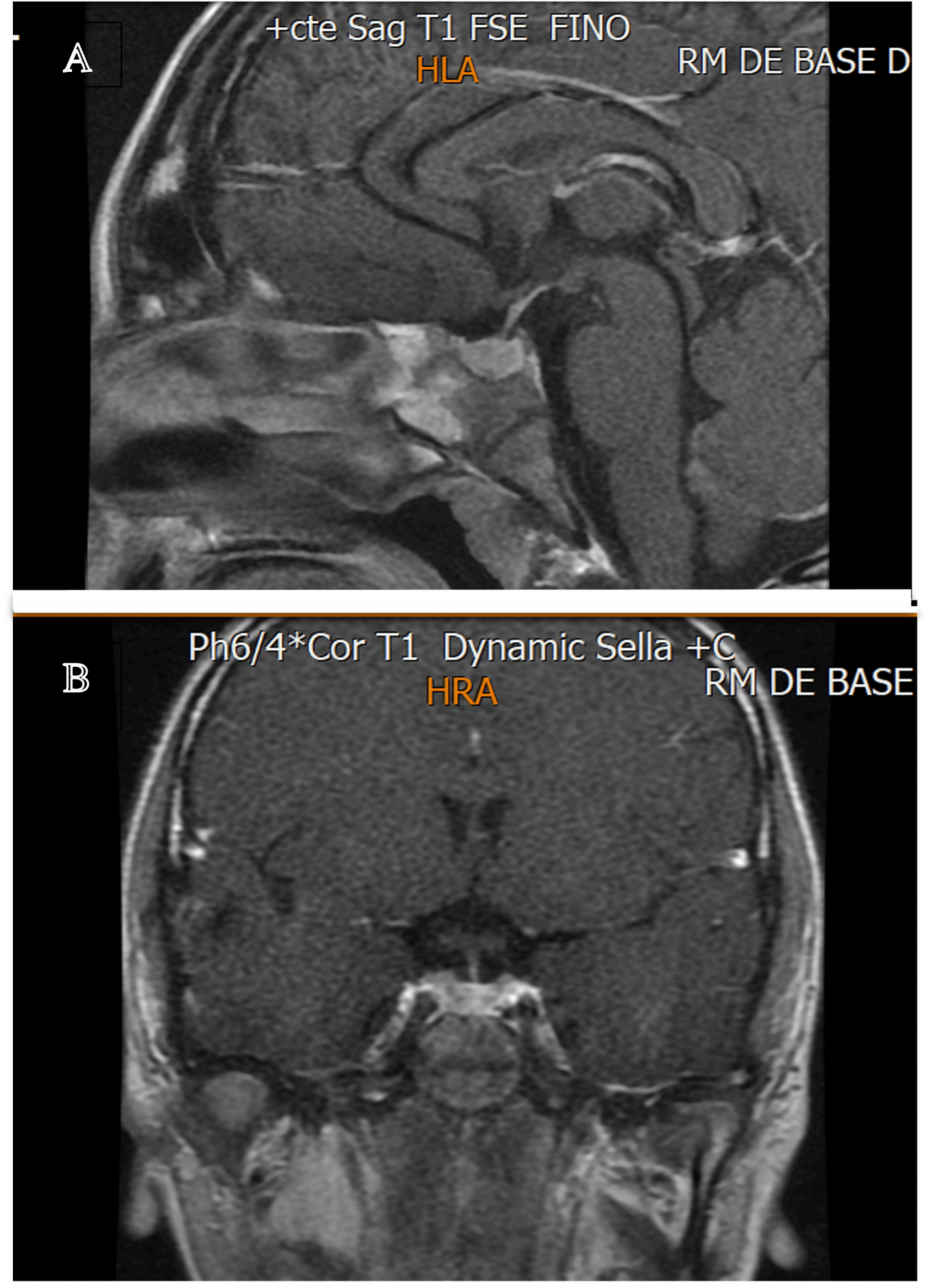
The MRI of the pituitary gland obtained three months after treatment showed regression of the enlargement and restoration of normal size (A) sagittal view; (B) coronal view

Once TSH and free T4 levels were within normal limits (TSH 4.8 mIU/L, free T4 1.2 ng/dL), a follow-up MRI of the sella turcica was performed three months later (Figure 2). It showed a completely normal pituitary gland and no evidence of pituitary enlargement, initially interpreted as an adenoma. No adverse events were reported during levothyroxine therapy.

Posterior case review with radiology and neurosurgery teams identified pituitary hyperplasia secondary to thyroid dysfunction, mimicking the appearance of a pituitary adenoma. The patient remained on thyroid hormone replacement, and TSH and free T4 levels were monitored periodically; however, no dosage adjustment was required.

## Discussion

Primary hypothyroidism is relatively common in children, with Hashimoto’s autoimmune thyroiditis being one of the leading causes. The presentation is often non-specific until hypothyroidism develops, with short stature and reduced growth velocity as the main clinical manifestations along with delayed puberty in some cases [5]. If primary hypothyroidism is not detected and treated promptly, chronic TRH drive producing increased TSH secretion can lead to pituitary hyperplasia mimicking a pituitary adenoma. The degree of pituitary hyperplasia depends on the duration and severity of hypothyroidism, as well as on the individual sensitivity of the pituitary to TRH. According to the medical literature, this variability is explained by the fact that pituitary hyperplasia is more frequently observed in cases of severe and prolonged hypothyroidism, where TRH stimulation is sustained [3]. The literature also suggests that children in stages of active growth may be more susceptible to pituitary hyperplasia secondary to hypothyroidism, possibly due to the greater plasticity and proliferative capacity of the gland during childhood [6,7]. In some cases, the presence of autoimmune thyroiditis may be associated with greater thyroid dysfunction and, consequently, increased pituitary stimulation [6].

Pituitary gland enlargement in children has a broad differential diagnosis. It may result from physiological hypertrophy during puberty (more commonly in adolescent females), pituitary hyperplasia secondary to primary hypothyroidism, pituitary adenomas (including those associated with gigantism), or non-adenomatous sellar and parasellar lesions such as germinomas, Langerhans cell histiocytosis, lymphocytic infundibuloneurohypophysitis, and Rathke’s cleft cysts [8-10]. As with all pituitary lesions, a complete evaluation of pituitary function is required [11]. It is important to note that, although adrenal insufficiency is theoretically possible if the pituitary hyperplasia compromises corticotroph cell function and reduces adrenocorticotropic hormone (ACTH) secretion, clinically significant ACTH deficiency is exceedingly rare. A more critical consideration is the concept of adrenal insufficiency being masked due to severe hypothyroidism, and it gets manifested when thyroxine is started due to rapid excretion of cortisol (improved glomerular filtration rate due to T4 correction); hence, cortisol insufficiency should be corrected (if present) before initiating thyroxine [12].

If this condition is suspected, pituitary surgery should be deferred [13, 14], as patients usually respond favorably to medical treatment with levothyroxine. Once thyroid function tests normalise, a follow-up MRI of the sella turcica should be performed to reassess pituitary size [15]. Once thyroid function tests normalise, a follow-up MRI of the sella turcica should be performed to reassess pituitary size [15]. Based on our findings, we recommend performing this follow-up imaging promptly to confirm the regression of hyperplasia, suggesting that three months post-therapy is the optimal window for reassessment. This process should be carried out in coordination with pediatric neurosurgery, thereby avoiding unnecessary pituitary surgery, which may result in complications such as hypopituitarism. Surgery is reserved for exceptional situations, such as the presence of mass effect symptoms or suspicion of a pituitary adenoma that does not regress with hormonal treatment. Therefore, although surgical cases have been reported in the literature, these instances are usually related to diagnostic errors or severe neurological compression [14].

## Conclusions

This case underscores the importance of thyroid function testing in all children presenting with pituitary enlargement before considering invasive procedures. If hypothyroidism is detected, thyroid hormone replacement should be initiated after first ruling out adrenal insufficiency. A follow-up pituitary MRI should then be performed once thyroid function tests have normalized, in order to reassess pituitary size. We recommend performing this imaging reassessment at three months post therapy. Heightened awareness of this entity is particularly important in resource-limited settings where diagnostic delays and misinterpretation of imaging remain common.
